# Case Report: Sequential postzygotic *HRAS* mutation and gains of the paternal chromosome 11 carrying the mutated allele in a patient with epidermal nevus and rhabdomyosarcoma: evidence of a multiple-hit mechanism involving *HRAS* in oncogenic transformation

**DOI:** 10.3389/fgene.2023.1231434

**Published:** 2023-08-10

**Authors:** Roberta Zuntini, Chiara Cattani, Lucia Pedace, Evelina Miele, Stefano Giuseppe Caraffi, Stefano Gardini, Elena Ficarelli, Simone Pizzi, Francesca Clementina Radio, Angelica Barone, Simonetta Piana, Patrizia Bertolini, Domenico Corradi, Maria Marinelli, Caterina Longo, Alberico Motolese, Orsetta Zuffardi, Marco Tartaglia, Livia Garavelli

**Affiliations:** ^1^ Medical Genetics Unit, Azienda USL, IRCCS, Arcispedale Santa Maria Nuova, Reggio Emilia, Italy; ^2^ Department of Pediatric Hematology, Oncology and Cellular and Gene Therapy, Ospedale Pediatrico Bambino Gesù, IRCCS, Rome, Italy; ^3^ Dermatology Unit, Azienda USL, IRCCS, Arcispedale Santa Maria Nuova, Reggio Emilia, Italy; ^4^ Molecular Genetics and Functional Genomics Research Unit, Ospedale Pediatrico Bambino Gesù, IRCCS, Rome, Italy; ^5^ Paediatric Hematology Oncology Unit, University Hospital of Parma, Parma, Italy; ^6^ Department of Oncology and Advanced Technologies, Pathology Unit, Azienda USL, IRCCS, Arcispedale S Maria Nuova, Reggio Emilia, Italy; ^7^ Department of Medicine and Surgery, Unit of Pathology, University of Parma, Parma, Italy; ^8^ Department of Dermatology, University of Modena and Reggio Emilia, Modena, Italy; ^9^ Department of Oncology and Advanced Technologies, Unit of Dermatology, Azienda USL, IRCCS, Arcispedale S Maria Nuova, Reggio Emilia, Italy; ^10^ Department of Molecular Medicine, University of Pavia, Pavia, Italy

**Keywords:** epidermal nevus, rhabdomyosarcoma, *HRAS*, postzygotic mutation, paternal UPD11

## Abstract

We report a 7-year-old boy born with epidermal nevi (EN) arranged according to Blaschko’s lines involving the face and head, right upper limb, chest, and left lower limb, who developed a left paratesticular embryonal rhabdomyosarcoma at 18 months of age. Parallel sequencing identified a gain-of-function variant (c.37G>C, p.Gly13Arg) of *HRAS* in both epidermal nevus and tumor but not in leukocytes or buccal mucosal epithelial cells, indicating its postzygotic origin. The variant accounted for 33% and 92% of the total reads in the nevus and tumor DNA specimens, respectively, supporting additional somatic hits in the latter. DNA methylation (DNAm) profiling of the tumor documented a signature consistent with embryonal rhabdomyosarcoma and CNV array analysis inferred from the DNAm arrays and subsequent MLPA analysis demonstrated copy number gains of the entire paternal chromosome 11 carrying the mutated *HRAS* allele, likely as the result of paternal unidisomy followed by subsequent gain(s) of the paternal chromosome in the tumor. Other structural rearrangements were observed in the tumours, while no additional pathogenic variants affecting genes with role in the RAS-MAPK and PI3K-AKT-MTOR pathways were identified. Our findings provide further evidence of the contribution of “gene dosage” to the multistep process driving cell transformation associated with hyperactive HRAS function.

## Introduction

The first description of a patient with a “pigmented patch” forming a linear pattern running exactly down the midline of the body, from the forehead to the groin, dates back to 1945 (Zlotnikoff, 1945). Epidermal nevi (EN) are developmental abnormalities in epidermal proliferation and are typically noted at birth or within the first year; they are related but distinct from sebaceous organoid nevi, although the two entities frequently coexist. The linear pattern often follows Blaschko’s lines, which are believed to represent dorsoventral migration patterns of neuroectodermal precursor cells during embryogenesis (Aslam et al., 2014). EN are estimated to occur in 1-3:1,000 live births, with males and females being equally affected (Garcia-Vargas et al., 2008). Although EN may occur as isolated findings, extensive evidence indicates that they are commonly associated with heterogeneous clinical features (Groesser et al., 2013), representing mosaic forms of genetically inherited conditions, as a result of postzygotic activating variants affecting genes coding for signal transducers positively controlling the RAS-MAPK and PI3K-AKT-MTOR signalling cascades (Hafner et al., 2006; Hafner et al., 2007; Hafner et al., 2012; Groesser et al., 2013). Both pathways play a key role in the control of multiple cellular processes, including proliferation, survival and energetic metabolism (Gadea et al., 2020), and their dysregulation represents a common event in cancer. Pediatric patients with widespread distribution of EN developing tumours (e.g., rhabdomyosarcomas, urothelial carcinomas, and squamous cell carcinomas) at young ages have been reported (Bourdeaut et al., 2010; Brandling-Bennett and Morel, 2010; Hafner et al., 2011). Among these, the onset of rhabdomyosarcoma in patients with EN harbouring somatic mutations in *KRAS* has been described (Om et al., 2017; Gadea et al., 2020; Chang et al., 2021).

Here, we report on a pediatric patient showing a diffuse EN at birth associated with a postzygotic activating missense variant in *HRAS* (p.Gly13Arg), who developed a left paratesticular embryonic rhabdomyosarcoma. Loss of the maternal chromosome and gains of the entire paternal chromosome 11 carrying the mutated *HRAS* allele, was detected in the tumour sample. This finding provides further evidence of the gene dosage effect contributing to HRAS-driven oncogenesis.

## Materials and methods

### Parallel sequencing analysis

Genomic DNA was extracted from the skin biopsy, peripheral blood, buccal brush and rhabdomyosarcoma tissue of the patient, and parallel sequencing was performed on them using a panel comprising 250 genes associated with tumor predisposition and progression, looking for mutations.

A panel including 250 genes associated with tumor predisposition and progression was scanned for mutations. Target enrichment and library preparation were performed using a custom-designed SeqCap EZ Hyper Cap Library assay (ROCHE, Pleasanton, CA, United States), and sequencing was performed on an Illumina NextSeq550 platform (Illumina, San Diego, CA, United States). A previously described in-house bioinformatics pipeline was used for data analysis (Lin et al., 2021; Radio et al., 2021). Reads alignment against the UCSC hg19 reference genome and variant calling (HaplotypeCaller v3.7) were attained following the GATK best practices (Van der Auwera et al., 2013). Variant annotation included occurrence in public (gnomAD) and in-house population databases, predicted functional relevance [e.g., CADD v1.4 (Kircher et al., 2014), M-CAP v1.3 (Jagadeesh et al., 2016)], and classification according to the American College of Medical Genetics and Genomics criteria (InterVar v2.0.1). Mean target coverage (>400x) was set to allow the identification of both germline and somatic events, and trio genotyping made it possible to infer allele transmission of variants of interest.

### DNA methylation profiling microarray analysis

DNA methylation (DNAm) profiling was performed using the Illumina Infinium Human Methylation EPIC Bead Chip (EPIC) arrays (Illumina, San Diego, CA, United States) according to the manufacturer’s instructions, as previously reported (Miele et al., 2020). Tumour areas with the highest tumour cell content (≥70%) were selected from the formalin-fixed paraffin embedded (FFPE) tissue slides for DNA extraction. 250 ng genomic DNA were used as input material. The generated DNAm data were compared with the Heidelberg sarcoma classifier v12.2 (Koelsche et al., 2021) to assign a subgroup score for the tumour against the identified 62 different sarcoma methylation classes. The same array data were used to identify copy number alterations as previously described (Capper et al., 2018). Integrative Genomic Viewer (IGV) was used for graphical visualization of structural rearrangements and mapping genes onto regions of interest.

### MS-MLPA and microsatellite analysis

Complementary approaches were adopted to characterize the structural rearrangements involving chromosome 11 and the *HRAS* gene using genomic DNA obtained from patient’s peripheral blood leukocytes, nevus and tumour samples. First, methylation-specific multiplex ligation-dependent probe amplification (MS-MLPA) was carried out using the MS-MLPA probe (ME030 C3-0121, MRC-Holland, Amsterdam, Netherlands) to confirm the copy number changes and analyze the CpG island methylation status of the 11p15 region. The assay was performed according to the manufacturer’s instructions. The products were run on an ABI 3500 Dx genetic analyzer (Applied Biosystems) and then analyzed using Coffalyser.net software (v.210,604.1451).

The evaluation of the parental origin of the mutated *HRAS* allele and duplicated chromosome 11 was evaluated on DNA extracted from patient’s peripheral blood leukocytes, nevus and tumour samples and his parents (peripheral blood leukocytes) through the analysis of fourteen microsatellites (D11S4046, D11S1338, D11S902, D11S904, D11S935, D11S905, D11S4191, D11S1314, D11S901, D11S4175, D11S925, D11S4151, D11S1320, and D11S968; panel 13 Thermo Fisher Scientific, Waltham, Massachusetts, United States) by automated sequencing.

## Results

### Clinical phenotype

**FIGURE 1:**
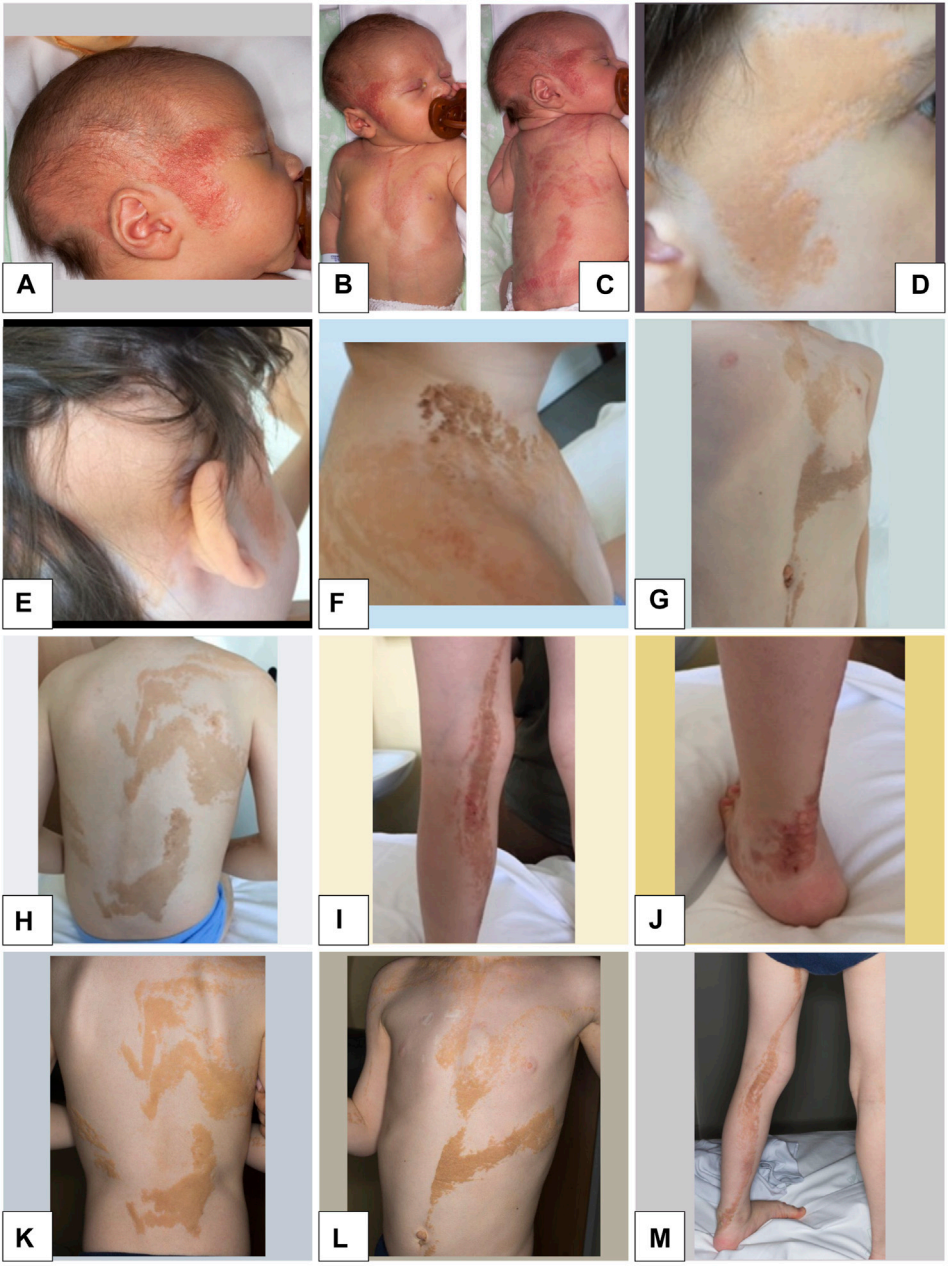
Epidermal nevi in the proband. **(A–C)**: At Birth: diffuse epidermal nevi (EN) on the face and head, right upper limb, thorax, left lower limb, arranged according to Blaschko’s lines. **(D–M)**: Age 7 years 2 months: diffuse EN of the face and head, right upper limb, thorax, left lower limb with rather irregular margins, partially confluent and with linear disposition to the limbs according to Blaschko’s lines of pink-orange color.

The boy was the first child of healthy, non-consanguineous parents. The pregnancy was complicated by hypothyroidism in the mother, who was not exposed to any known teratogens. Fetal movements were described as normal. Prenatal ultrasound scans were normal. The proband was born at 40 weeks of gestation, delivered by Caesarean section due to dynamic dystocia. At birth, weight was 3,610 g (25th percentile), length was 49 cm (85th percentile), and head circumference was 36.5 cm (97th percentile). Apgar scores were 9 at 1 min and 10 at 5 min, respectively. He was admitted to the neonatal department due to diffuse proliferation of EN on the face and head, right upper limb, thorax, and left lower limb (Figures 1A–C).

Psychomotor development was normal, with head control at 2 months, sitting without support at 6 months, autonomous walking at 18 months, first words at 18 months. At present, he attends primary school with good grades.

At the age of 18 months he developed a left paratesticular embryonic rhabdomyosarcoma, for which he was operated and underwent standard chemotherapy treatment of vincristine and actinomycin-D (Arndt et al., 2009; Oberlin et al., 2012).

At last clinical examination (7 years 2 months), height was 115.5 cm (10th percentile), weight was 17.5 Kg (<third percentile), and head circumference was 51 cm (25th-50th percentile). Pubertal stages (Tanner) were A0 P1 B1, and volume of the right testicle was 1–2 mL. He had high forehead in absence of facial dysmorphisms. He showed diffuse EN of the face and head, right upper limb, thorax, and left lower limb, with rather irregular margins, partially confluent and with linear disposition to the limbs according to Blaschko’s lines of pink-orange colour (Figures 1D–M). Some lesions were more prominent and browner in the neck and ankle, where they appeared thickened, particularly at the sites of microtrauma (Figures 1F, J).

Transfontanellar ultrasound, abdominal ultrasound, audiometric evaluation including ABR, brain MRI, abdominal MRI, EEG and ophthalmological evaluation were all normal. Echocardiography revealed mild patent foramen ovale.

**FIGURE 2:**
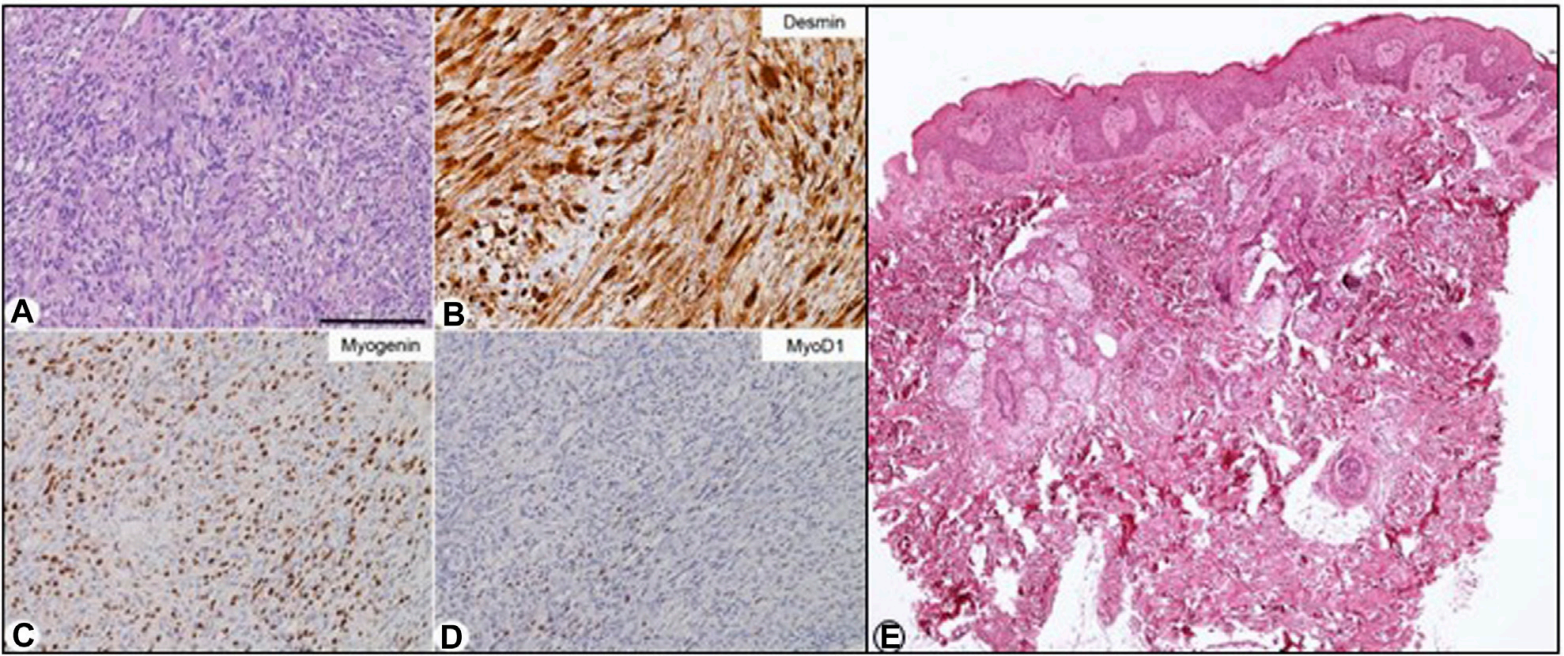
Biopsy and histological examination of the left paratesticular embryonic rhabdomyosarcoma. **(A–D)**: Histopathology. **(A)** Medium-power histopathological view of this tumour, which consisted of a number of atypical spindled cells with eosinophilic cytoplasms. In this setting, occasional ganglion-like cells were present in some places. The tumour cells were immunohistochemically decorated by an anti-desmin antibody **(B)**, anti-myogenin [**(C)**; about 60% of the cells], and anti-MyoD1 [**(D)**; about 10%–20% of the cells] antibodies. Stainings: **(A)** hematoxylin-eosin; **(B–D)**: Immunohistochemical reactions revealed by 3,3′-Diaminobenzidine (DAB) and mildly counterstained by Harris hematoxylin. **(E)** Skin biopsy: histological examination of the skin of the back compatible with epidermal nevus: Mild irregular hyperplasia and focal hyperkeratosis of the epidermis and few inflammatory cells in the superficial dermis. Original magnification: **(A–D)**: ×20 (bar is 250 μm), **(E)** EEx40.

The histological examination of the paratesticular lesion revealed a malignant ectomesenchymoma consisting of embryonic spindle cell-variant rhabdomyosarcoma with neuroectodermal differentiation component of ganglioneuroma type (Figures 2A–D). RT-PCR analysis was performed to assess *MyoD1* expression, which was positive, and *PAX3*-*FOX01*, *PAX7*-*FOX01, and PAX3*-*NCOA1* rearrangements, which were negative.

The histopathological examination of a skin biopsy from the patient’s back documented irregular hyperplasia of the epidermis, acanthosis, and hyperkeratosis, consistent with EN histology (Figure 2E).

### Molecular analyses

**FIGURE 3:**
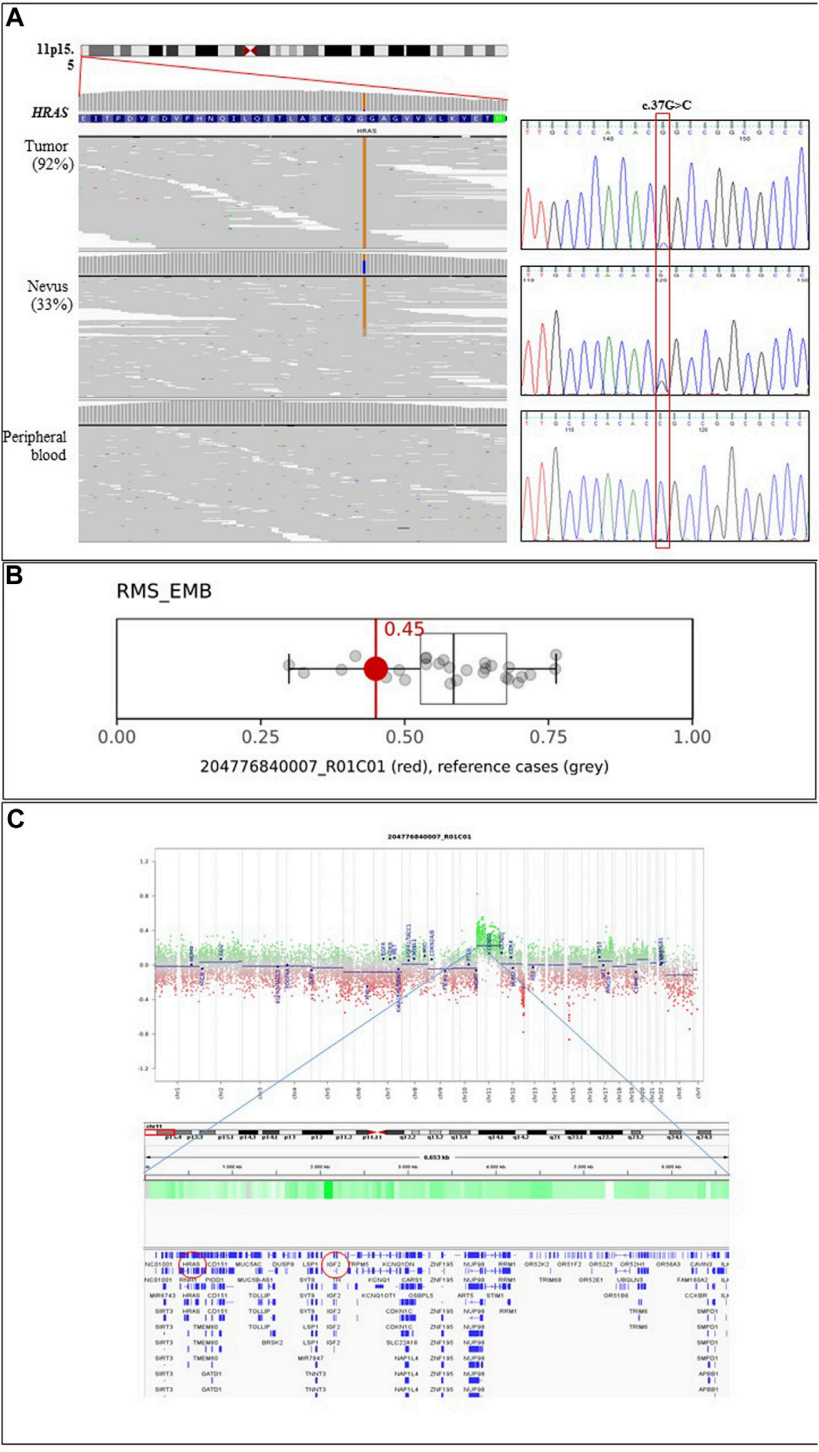
Molecular analyses. **(A)** The c.37G>C missense variant (p.Gly13Arg) in *HRAS* is found at the homozygous state in the tumour specimen of the patient, while it is observed as a heterozygous change in the epidermal nevus of the same subject. Parallel sequencing output of the portion of the first coding exon of the *HRAS* gene (ENST00000311189.7) is shown on the left. The variant is not observed in leukocytes. The panels show the reads alignments of three tested tissues. The variant accounted for 33% and 92% of total reads in the EN and tumour, respectively. Chromatograms from Sanger sequencing validation are reported on the right. Absence of the variant in peripheral blood documents the somatic origin of the variant. **(B)** Box-and-whisker plots depicting the maximum raw classification scores (0.45) of the tumour sample in the methylation class “Rhabdomyosarcoma, Embryonal (RMS_EMB)” according to Sarcoma Classifier v12.2 (https://www.molecularneuropathology.org/mnp/classifier/9). Grey dots represent the reference cases in the methylation class. **(C)** ERMS Copy Number Variation plots (upper panel) inferred from DNAm data and IGV snapshot (lower panel) detailing the genes mapping in the indicated duplicated regions. Red circles highlight *HRAS* and *IGF2* genes.

Parallel sequencing directed to scan a selected panel of genes using genomic DNA obtained from the skin lesions, blood, buccal brush and rhabdomyosarcoma tissue samples was performed, allowing the identification of a somatic pathogenic missense variant in the *HRAS* gene (c.37G>C, p.Gly13Arg; NM_005343.4) in both the epidermal nevus and tumour tissues. Variant reads accounted for 33% and 92% of total reads in the nevus and tumour, respectively, supporting the loss of the wild-type allele in the latter (Figure 3A). The variant was not detected in the DNA samples extracted from blood and buccal brush, indicating its postzygotic origin.

DNAm microarray analysis of the proband’s tumour sample provided a profile that was consistent with the episignature characterizing embryonic rhabdomyosarcomas, showing a calibrated score of 0.99 (Figure 3B and Supplementary Table S1), in line with the pathological findings. Copy number variation analysis documented a gain of the entire chromosome 11, indicating duplication events involving the chromosome carrying the mutated *HRAS* allele (Figure 3C). Two genetic losses were also observed, involving chromosomes 12 (q24.21-q24.33; chr12:120,182,403_133,841,900) and 15 (q12-q13.2; chr15:28,418,871_30,574,776) (Supplementary Figure S1). The deletion on chromosome 12 involves 184 annotated genes, whose germline pathogenic variants have been associated with neurodevelopmental disorders; to date, this region has not been reported to substantially contribute to oncogenesis. The loss on chromosome 15 contains 36 genes, and considering both germline and somatic events, no information is available on its involvement in the tumourigenesis.

**FIGURE 4:**
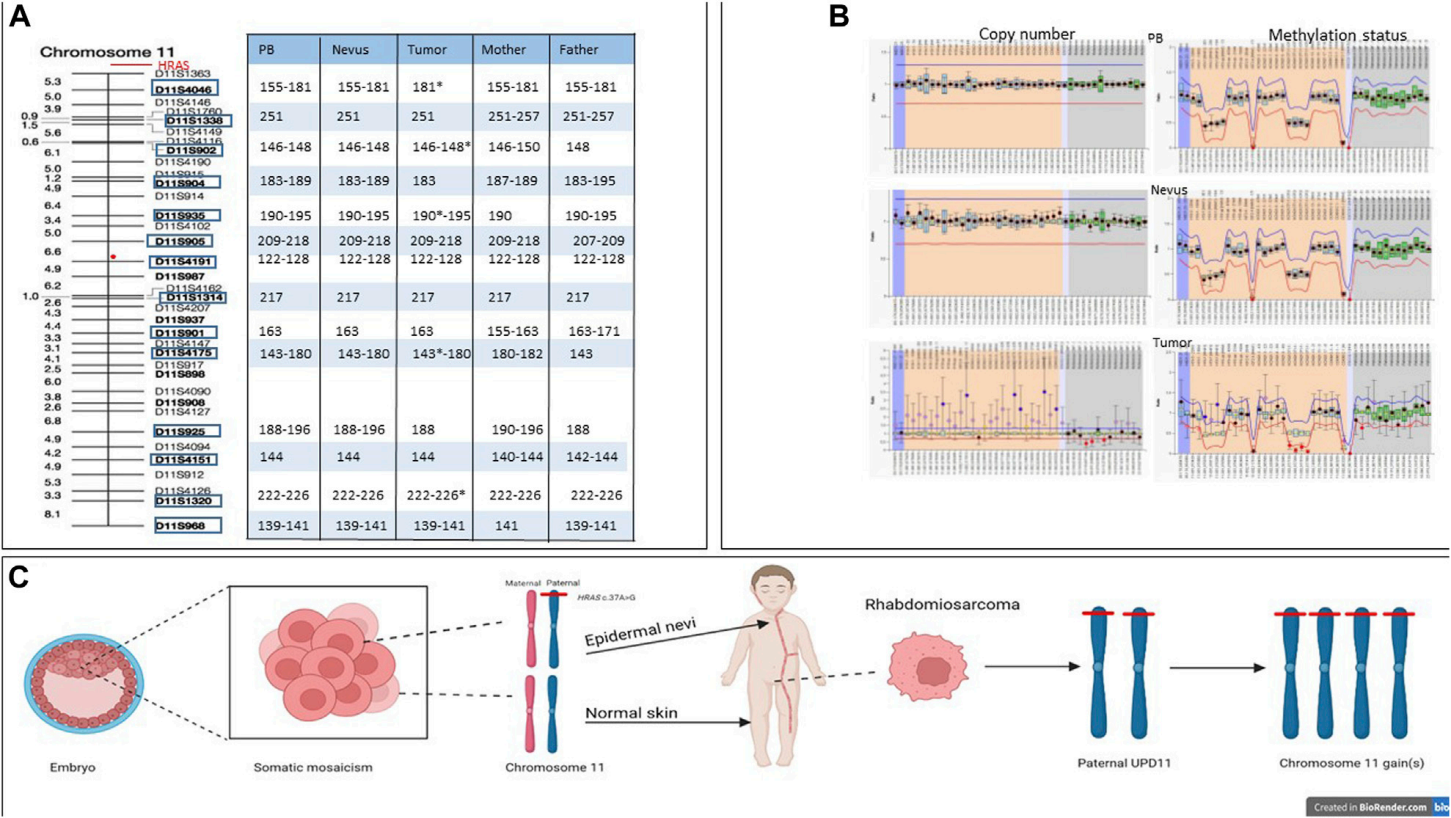
Microsatellite and MLPA analyses and multi-hit model in HRAS-associated neoplastic transformation. **(A)** Microsatellite analysis of patient’s peripheral blood normal and tumor tissues and peripheral blood of his parents, showing length of each marker for each tissues. The red circle indicates the centromere position. Stars indicate alleles with increased copy number. **(B)** MLPA analysis of normal tissue (upper plot), nevus (middle plot) and tumor tissue (lower plot). **(C)** The c.37G>C missense variant (p.Gly13Arg) arose postzygotically in the paternal *HRAS* allele, resulting in diffuse EN along Blaschko’s line. Later, in the left paratestis, paternal unidisomy of chromosome 11 and subsequent gain(s) of the same chromosome carrying the mutated *HRAS* allele is hypothesized.

Chromosome 11 contains two clusters of imprinted genes located in the 11p15.5 region regulated by separate Imprinting Control regions (IC1 and IC2). To assess the parental origin of the somatic mutation we first performed the MS-MLPA analysis, which showed an altered methylation index of 0.93 on IC1 and a methylation index of 0.12 on IC2 (Figure 4A), which was consistent with an amplification of the paternal chromosome as reported by Jiang et al. (2021). This finding was confirmed by microsatellite analysis (Figure 4B). Overall, the molecular analyses support a complex series of somatic events involving the *HRAS* allele of the paternal chromosome 11 consisting of an intragenic activating mutation in the EN, a duplication of the mutated allele with loss of the wild-type allele possibly due to a parental unidisomy event, and subsequent gain(s) of the duplicated chromosome.

### Review of the literature

A systematic literature search was performed using PubMed (https://www.ncbi.nlm.nih.gov/pubmed; data freeze 28 March 2023) to identify reports on patients presenting with molecularly characterized EN by using the following search equations: (“epidermal nevi”) AND (“somatic mutation”); (“epidermal nevi”) AND (“HRAS”). One hundred and thirty-four papers, published between 2002 and 2023, were retrieved. Thirty-seven articles reported somatic variants in the *FGFR3*, *FGFR2*, *PIK3CA*, *KRAS*, and *HRAS* genes from 90 patients presenting with EN. Among these, 61 (68%) cases presented alterations in *RAS* genes. The gene variants, analyzed tissues and clinical features of the patients are summarized in Supplementary Table S2. Thirty-two patients (35%) had developed tumours, which were benign in 19 subjects (59%) and malignant neoplasms in 13 (41%). Among the 19 patients who developed benign tumours, only one (6%) carried somatic variant in *KRAS*, 16 (84%) showed a mutated *HRAS* allele, and two (10%) had mutations in *PIK3CA* genes; the 13 patients with cancer harboured somatic variants exclusively in the *KRAS* (31%) and *HRAS* (69%) genes*.*

## Discussion

We describe the second case of a somatic variant in *HRAS* (c.37G>C, p.Gly13Arg) causing EN and contributing to rhabdomyosarcoma, the latter process involving multiple gains of the entire paternal chromosome 11 carrying the mutated *HRAS* allele.

Rhabdomyosarcoma is the most common soft-tissue sarcoma of childhood (Ognjanovic et al., 2009). It is classified by histology into two main subtypes, alveolar (ARMS) and embryonal (ERMS), having distinct molecular and clinical profiles. Somatic mutations in *RAS* genes are frequently found in EN and are also involved in the onset of ERMS, being observed in approximately 25% of the cases (Agaram et al., 2022). To date, five patients carrying somatic mutation in codon 35 of *KRAS* and codon 13 of *HRAS* with extensive EN and rhabdomyosarcoma have been reported (Bourdeaut et al., 2010; Om et al., 2017; Chang et al., 2021; Luo et al., 2021; Davies et al., 2022). Two additional patients have been described without molecular characterization (Rongioletti and Rebora, 1991; Vidaurri-de la Cruz et al., 2004). RAS proteins are molecular switches controlling the activation of several signal transduction pathways, most of which supporting cell proliferation and survival, and are commonly mutated in cancer (Bos, 1989). Germline mutations in these genes also underlie a well-defined group of developmental disorders collectively known as “RASopathies”, which are characterized by variable cancer predisposition (Tartaglia and Gelb, 2010). Among the RASopathies, a greater risk of developing malignancy characterizes patients with Costello Syndrome (CS), with a 15% cumulative risk of cancer at age 20, generally represented by RMS, neuroblastoma and bladder cancer (Zampino et al., 2007; Dunnett-Kane et al., 2020). More than 90% of the mutations in CS patients are clustered in codons 12 and 13 (p.Gly12Ala/Ser/Val/Cys/Asp/Glu and p.G13Cys/Asp), constituting a mutational hotspot (Giannoulatou et al., 2013). Similarly, 98% of the *RAS* gene mutations implicated in cancer involve Gly12, Gly13 and Gln61 (COSMIC database, https://cancer.sanger.ac.uk/cosmic) (COSMIC, 2023), which are key residues for the intrinsic and GAP-mediated RAS GTPase activity, and when mutated result in constitutively active proteins. The somatic p.Gly13Arg substitution in HRAS is frequently found in cancer and EN, while it has not been described in CS patients, suggesting that it may be tolerated only in a mosaic state, resulting lethal as a germline event (Hafner et al., 2012). Of note, while patients with variants involving the PI3K-AKT pathway have been associated with benign neoplasms (e.g., lipomas and trichoblastomas), malignancies (e.g., rhabdomyosarcomas and bladder cancer) have exclusively been reported in patients with EN associated with somatic variants of *RAS* genes (Supplementary Table S2).

We detected a variant allele frequency (VAF) of 33% in the EN. The same variant was not detected in blood and buccal brush samples, supporting its post-zygotic origin, in line with previous reports (Supplementary Table S2). The same variant showed an increased VAF (92%) in the tumor, which is in line with previous findings reported in Costello Syndrome indicating a second somatic hit generally associated with uniparental disomy (UPD) (Kratz et al., 2007). CNV and MLPA analyses performed on the tumor tissue, however, indicated the occurrence of additional entire chromosome 11 gain(s) (Figure 3C and Figure 4B), which had not previously been observed (Hafner et al., 2011; Parham and Barr, 2013; Avitan-Hersh et al., 2014; Li et al., 2014; Farschtschi et al., 2015; Om et al., 2017; Gadea et al., 2020), further indicating a role of a dosage effect for the mutated *HRAS* allele in tumorigenesis.

Gain of chromosome 8 represents the most common cytogenetic alteration described in ERMS (70%). Additional recurrent structural rearrangements involve chromosomes 2, 7, 11, 12, 13, 20 (gains, 25%–50%) and chromosomes 9 and 10 (losses, 20%–30%) (Bourdeaut et al., 2010; Parham and Barr, 2013; Clay et al., 2021). However, a major hallmark of ERMS is the occurrence of UPD at 11p15.5, a region that contains *HRAS* and a cluster of imprinted genes (Kratz et al., 2007; Robbins et al., 2016), including *IGF2* (Figure 3C), whose increased RNA expression is a characteristic of these tumors. The molecular mechanisms underlying *IGF2* overexpression can be due to either loss of heterozygosity (LOH) or loss of imprinting (LOI) at the 11p15.5 locus (Martins et al., 2011). In our patient, we demonstrated that the activating *HRAS* mutation arose on the paternal allele and the consequent gain(s) of that chromosome was possibly the result of an UPD with loss of the maternal allele followed by subsequent gain(s) of the paternal chromosome, the latter representing an additional hit contributing to neoplastic transformation (Figure 4C). Unfortunately, no information on the parental origin of the *HRAS* alleles carrying the pathogenetic variants in the previously reported cases is available.

As reported by Hobbs et al. (2016) and in the COSMIC database (COSMIC, 2023), different mutations in *RAS* genes are associated with predisposition to specific tumors, therefore molecular analysis is recommended in newborns presenting diffuse EN to detect pathogenic variants. The risk of cancer in patients harboring somatic mutations depends on the stage of embryonic development in which the variant occurs and therefore on the number and type of tissues involved. Although the incidence and the individual risk of tumors in patients with EN is not known, it is conceivable that patients with EN might have an increased risk of non-cutaneous malignancies of various organs during life; therefore, it would be advisable to follow these patients periodically in order to limit the risk of carcinogenesis from early childhood. In line with these considerations, the present findings outline the importance of a careful follow-up of these patients requiring periodic oncohematological and dermatological evaluations, urine tests, and kidney, urinary tract and thyroid ultrasound. Furthermore, given the high incidence of cardiac anomalies (60%–90%) associated with germline RASopathies (Calcagni et al., 2020), cardiac evaluation with echocardiogram at least at the time of diagnosis might be also indicated in these patients. We appreciate that, while the development of therapies targeting RAS proteins has been challenging (Moore et al., 2020; Conroy et al., 2021), new opportunities resulting from the development of inhibitors that are able to target RAS proteins in their active or inactive state are emerging, and therapeutic strategies for targeting oncogenic RAS are currently explored in clinical trials (Moore et al., 2020; Punekar et al., 2022), making even more relevant the identification of the driver somatic variants for a more effective surveillance and care.

## Data Availability

The datasets presented in this study can be found in online repositories. The names of the repository/repositories and accession number(s) can be found below: https://www.ncbi.nlm.nih.gov/snp/, rs104894228.
