# Supplementary material for: Case Report: Sequential postzygotic HRAS mutation and gains of the paternal chromosome 11 carrying the mutated allele in a patient with epidermal nevus and rhabdomyosarcoma: evidence of a multiple-hit mechanism involving HRAS in oncogenic transformation
Source: Front Genet. 2023 Aug 10;14:1231434. doi: 10.3389/fgene.2023.1231434 (PMC10447906; doi:10.3389/fgene.2023.1231434)
Supplement: Supplementary file 1 [file Table1.DOCX]

**Supplementary Table 1. Global DNA methylation calibrated scores of the index case in the recognized 65 methylation classes according to Sarcoma Classifier v12.2 (**[**https://www.molecularneuropathology.org/mnp/classifier/9**](https://www.molecularneuropathology.org/mnp/classifier/9)**). The index case reached an optimal classification score (> 0.99) in the methylation class “Rhabdomyosarcoma, Embryonal (ERMS)”.**

**
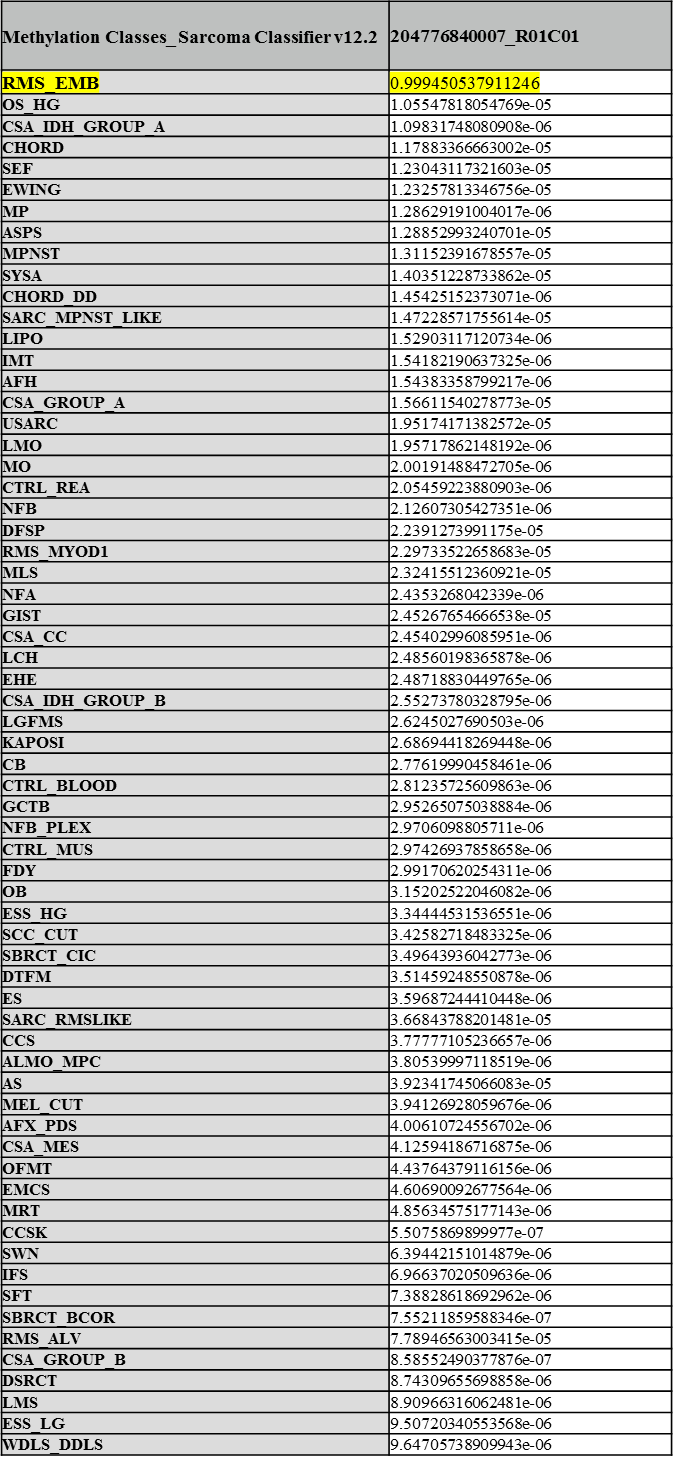
**
