# Supplementary figures and images for: Case Report: Sequential postzygotic HRAS mutation and gains of the paternal chromosome 11 carrying the mutated allele in a patient with epidermal nevus and rhabdomyosarcoma: evidence of a multiple-hit mechanism involving HRAS in oncogenic transformation
Source: Front Genet. 2023 Aug 10;14:1231434. doi: 10.3389/fgene.2023.1231434 (PMC10447906; doi:10.3389/fgene.2023.1231434)

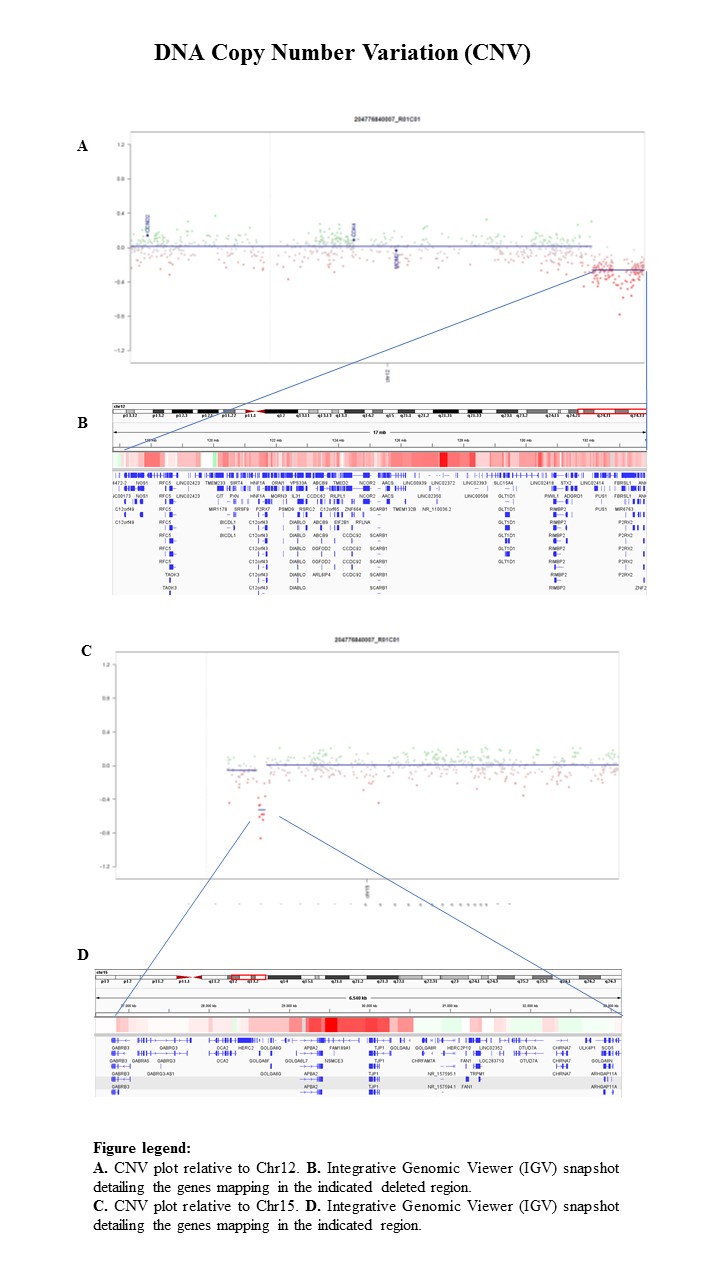

Supplement: Supplementary file 2 [file Image1.JPEG]
