# Supplementary material for: Case Report: Sequential postzygotic HRAS mutation and gains of the paternal chromosome 11 carrying the mutated allele in a patient with epidermal nevus and rhabdomyosarcoma: evidence of a multiple-hit mechanism involving HRAS in oncogenic transformation
Source: Front Genet. 2023 Aug 10;14:1231434. doi: 10.3389/fgene.2023.1231434 (PMC10447906; doi:10.3389/fgene.2023.1231434)
Supplement: Supplementary file 3 [file Table2.docx]

| **Supplementary Table 2. Literature review reporting somatic variants in the genes involved in EN.** | | | | | | |
| --- | --- | --- | --- | --- | --- | --- |
| **Case** | **Sex** | **Age** | **Clinical features** | **Tissue analysed**  **(% mutant allele burden)** | **Gene mutations** | **References** |
|  | M | Newborn | EN along Blaschko’s lines, r**habdomyosarcoma** | EN (33%), rhabdomyosarcoma (92%), buccal brushing (0%), blood (0%) | HRAS: c.37G>C, p.Gly13Arg | Our case |
| 1 | M | 23 | PPK, Trichoblastoma | Sebaceous nevus (~50%), melanocytic nevus (65%), keratinocytes (0%), hair (0%),buccal brushing (0%), blood (0%) | *HRAS:* c.37G>C, p.Gly13Arg | Wollenberg et al, 2002 |
| 2 | F | 12 yo | EN and mild facial dysmorphism | EN (27–29%), keratinocytes (34–38%), hair root (0%), buccal brushing (0%), blood (0%) | *FGFR3*: c.742C>T, p.Arg248Cys | Collin et al, 2007 |
| 3 | 8M/  6F | 14 pt (1- 65 yo) | EN | NA | *PIK3CA*: c.1624G>A, p.Glu545Gly  *FGFR3*: c.742C>T, p.Arg248Cys | Hafner et al, 2007 |
| 4 | F | 5 yo | EN, seizures, delayed language and psychomotor development, brain abnormalities | EN(~35%), normal skin (0%), blood (50%) | *FGFR3*: c.742C >T, p.Arg248Cys | Garcia-Vargas et al, 2008 |
| 5 | F | 38 yo | Schimmelpenning-Feuerstein-Mims  syndrome, Trichoblastoma | Nevi (NA), trichoblastoma (NA), blood (0%) | *HRAS:* c.37G>C, p.Gly13Arg | Wiedemeyer et al, 2009 |
| 6 | F | Newborn | EN along Blaschko’s lines, **rhabdomyosarcoma**, micropolycystic  kidneys and growth retardation | Epidermal component EN (~50%),  dermal component EN (0%), rhabdomyosarcoma (NA), normal skin (0%), blood (0%) | *KRAS*: c.35G>A, p.Gly12Asp | Bourdeaut et al, 2010 |
| 7 | F | 17 yo | EN along Blaschko’s lines, scoliosis | EN (~60%), intraoral EN (~50%), normal skin (0%), hair (0%), urine sediment (0%), blood (~20%) | *FGFR3:* c.742C>T, p.Arg248Cys | Bygum et al, 2011 |
| 8 | M | 19 yo | EN, **UCC of the bladder** | UCC (~50%), UCC lung metastasis (~50%), EN (~50%), normal urothelium (~10%), bladder muscle (0%), angiomas (~0%), blood (~10%) | *HRAS*: c.34G>A, p.Gly12Ser | Hafner et al, 2011 |
| 9 | F | 18 yo | PPK along Blaschko’s lines | Nevi (NA), normal skin (0%), hair (0%), blood (0%) | *HRAS:* c.37G>C, p.Gly13Arg | Chantorn et al, 2011 |
| 10 | F | 6 months | EN along Blaschko’s lines, seizures and  brain abnormalities | EN (~21%), normal skin (0%), urothelial cells (0%), blood (0%) | *FGFR3*: c.746C >G, p.Ser249Cys | Ousager et al, 2012 |
| 11 | F | / | PPK, Trichoblastoma, Endometrial polyp, **Basal Cell Carcinoma**, **UCC** | Nevi (NA), normal skin (0%), buccal brushing (0%), endometrial polyp (0%), Basal Cell Carcinoma (NA), trichoblastoma (NA), UCC (NA), blood (0%) | *HRAS:* c.37G>C, p.Gly13Arg | Groesser et al, 2013 |
| 12 | / | 14 pt (Newborn-17 yo)  11 yo  11 yo  11 pt (12-57 yo) | EN  EN  EN  EN, SCP, Trichoblastoma, TAA | EN (~10-50%), blood (0%)  EN (~33%), blood (0%)  EN (~45%), blood (0%)  EN (~25-50%), benign tumours (~25-50%), blood (0%) | *HRAS:* c.37G>C, p.Gly13Arg  *KRAS*: c.35G>T, p.Gly12Val  *KRAS*: c.35G>A, p.Gly12Asp  *HRAS:* c.37G>C, p.Gly13Arg | Levinsohn et al, 2013 |
| 13 | M | 14 yo | EN, hypotonia of the right arm, thymoma, cystic lesions in hand and  cervical bones | EN (~50%), thymoma (~50%), blood (0%) | *HRAS:* c.37G>C, p.Gly13Arg | Avitan-Hersh et al, 2014 |
| 14 | M | 21 yo | EN, multiple spinal tumours, lipoma, duplicated renal arteries, ectasia of aorta, scoliosis | Spinal tumours (3-7%), dermis (0%) | *PIK3CA*: c.3140A >T, p.His1047Leu | Keppler-Noreuil et al, 2015 |
| 15 | F | 18 yo | PPK along Blaschko’s lines with a combined **melanocytic and adnexal neoplasm** | Epithelial tumour (33%), melanocytic tumour (20%), blood (0%) | *HRAS:* c.37G>C, p.Gly13Arg | Li et al, 2014 |
| 16 | F  F  F  M | 5 yo  12 yo  15 yo  16 yo | EN, hypophosphatemia, skeletal dysplasia, brainstem lipoma, thyroid nodule, splenic haemangiomas  EN, hypophosphatemia, skeletal dysplasia, subaortic valve stenosis  EN, hypophosphatemia, skeletal dysplasia, eccrine poroma  EN, hypophosphatemia, skeletal dysplasia, colpocephaly, periventricular white matter paucity | EN (~30%), Blood (0%)  EN (~24%), Blood (0%)  EN (~38%), Blood (0%)  EN (~44%), Blood (0%) | *HRAS:* c.37G>C, p.Gly13Arg  *HRAS:* c.37G>C, p.Gly13Arg  *HRAS:* c.37G>C, p.Gly13Arg  *HRAS:* c.37G>C, p.Gly13Arg | Lim et al, 2014 |
| 17 | F  F | 10 yo  6 yo | PPK, woolly hair  Woolly hair nevus | EN (~55%), blood (8%)  EN (50%), curly hair (50%), straight hair (0%), blood (0%) | *HRAS*: c.34G>A, p.Gly12Ser  *HRAS*: c.34G>A, p.Gly12Ser | Levinsohn et al, 2014 |
| 18 | M | 14 yo | verrucous hyperpigmented skin lesions, **paravertebral conglomerate tumour**, intraspinal lipoma | hyperpigmented verrucous skin sample (44%), intraneural Schwann cell proliferation (49%), the lipoma sample (56%), blood (0%) | *KRAS*: c.35G>A, p.Gly12Asp | Farschtschi et al, 2015 |
| 19 | F  F  F  F | 5 yo  27 yo  12 yo  16 yo | KEN, twisted hair, right eye bulging.  KEN with comedos  KEN, macrocephaly, Arnold Chiari type II malformation, toe syndactyly, scoliosis, overgrowth of hands and feet, capillary malformation.  KEN along Blaschko’s lines | EN (35.85%), blood (3,37%)  EN (~42%), blood (0%)  EN (~25%), blood (0%)  EN (~36%), blood (0%) | *FGFR2*: c.1127A>G, p.Tyr376Cys *FGFR2*: c.755C>G, p.Ser252Trp  *FGFR2*: c.1127A>G, p.Tyr376Cys  *FGFR2:* c.857C>T, p.Pro286Ser | Toll et al, 2016 |
| 20 | F | 12 yo | PPK, severe rickets, and skeletal dysplasia | EN (~26%), saliva (0%) | *HRAS:* c.37G>C, p.Gly13Arg | Lim et al, 2016 |
| 21 | F | 3 yo | EN along Blaschko’s lines | EN (~40%), derma (~10), normal skin (0%), | *KRAS* c.34G>T, p.Gly12Cys | Igawa et al, 2016 |
| 22 | F | 5 months | PPK along Blaschko’s lines **vaginal botryoid rhabdomyosarcoma** | EN (~50%), SLN (0%), vaginal botryoid rhabdomyosarcoma (~50%), blood (0%) | *KRAS* c.35G>C p.Gly12Ala | Om et al, 2017 |
| 23 | M | 59 yo | EN, SK | EN (17,9%), SK (24,5%) | *HRAS:* c.37G>C, p.Gly13Arg | Kitamura et al, 2017 |
| 24 | M | 14 yo | EN along Blaschko’s lines, scleroderma-like lesion, melorheostosis | EN (31%), Scleroderma-like lesion (16%), normal skin (0%), blood (0%) | *KRAS:* c.183A >C, p.Gln61His | Whyte et al, 2017 |
| 25 | F | 2 yo | EN along Blaschko’s lines, bilateral cortical dysgyria | EN (30%), normal skin (0%), urine sediment (10%) hair (5%), saliva (5%), blood (0%) | *FGFR3:* c.746C>G. p.Ser249Cys | Bessis et al, 2017 |
| 26 | F | 18 yo | EN along Blaschko’s lines, woolly, hair and PPK | EN (~40%), woolly hair(~45%), normal hair (0%), blood (0%) | *HRAS*: c.34G>A, p.Gly12Ser | Honda et al, 2017 |
| 27 | M | 33 yo | PPK along Blaschko’s lines, localized curly hair, precocious puberty | affected skin (30%), unaffected skin (5%), saliva (9%), blood (4%) | *HRAS:* c.34G>C; p.Gly12Arg | Martin et al, 2018 |
| 28 | M | newborn | EN, lipoma, vascular malformation | EN (46%), lipoma (50%), vascular malformation (58%), buccal mucosa (24%), blood (0%) | *PIK3CA*: c.241G>A, p.Glu81Lys | Denorme et al, 2018 |
| 29 | F | 3 yo | KEN along Blaschko’s lines | EN (~41%), blood (0%) | *FGFR2:* c.1144T>C, p.Cys382Arg | Tanaka et al 2018 |
| 30 | M | 5 yo | EN, woolly hair nevus, linear pigmentation | EN (~28,6%), woolly hair (43,5%), blood (0%) | *HRAS:* c.34G>A, p.Gly12Ser | Nishihara et al, 2019 |
| 31 | /  F | 2 yo  7 yo | PPK, **Perioral squamous papilloma**, bone dysplasia  KEN along Blaschko’s lines, woolly hair nevus, bone dysplasia | skin (36%), blood (0%)  skin (34%), bone (10%), blood (0%) | *HRAS:* c.182A >T, p.Glu61Leu  *HRAS.* c.34G >T, p.Gly12Cys | Mestach et al, 2020 |
| 32 | M  F  F  F  F | young adult  child  child  young adult  adolescent | KEN, **Urothelial carcinomas,** twisted hair (right hemicranium)  KEN, macrocephaly, left hemibody overgrowth, extensive capillary malformation, foot syndactyly  KEN, twisted hair, proptosis  KEN  KEN | Skin (18%–31%), urothelial carcinomas (31%–49%), blood (14%)  Skin (23%), urine (2%–4%), blood (0%)  Skin (NA), urine (19%), blood (3.1%)  Skin (29%), urine (NA), blood (NA)  Skin (NA), urine (8%), blood (4%) | *HRAS:* c.34G>A, p.Gly12Ser  *PIK3CA:*c.248T>C, p.Phe83Ser  *FGFR2*: c.1127A>G, p.Tyr376Cys  *FGFR3:* c.742C>T, p.Arg248Cys  *HRAS:* c.35G>T, p.Gly12Val | Gadea et al, 2020 |
| 33 | M | 22 months | EN, asymmetric limb overgrowth, capillary malformation, **bilateral Wilms tumours** | EN (~50%), abnormal right kidney (40-60%), normal parenchyma of the left kidney (3-4%), blood (0%) | *KRAS:* c.35G>A, p.Gly12Asp | Slack et al, 2021 |
| 34 | M | 69 yo | EN, **Multiple squamous cell carcinomas** | EN (NA), SCC (NA) | *HRAS:* c.37G>C, p.Gly13Arg | Narahira et al, 76 |
| 35 | F  M | 5 yo  Newborn | EN, coarctation of the aorta, overgrowth  of the right leg, HPV-negative papilloma, developmental/dysplastic cystic kidney, benign right thyroid nodule,  hypertension, and allergic rhinitis.  EN along Blaschko’s lines, patent ductus arteriosus, low-set ears, fifth finger clinodactyly, aplasia cutis of the scalp pulmonary vascular dysplasia and hypoplasia, hepatosplenomegaly, **embryonal rhabdomyosarcoma** | EN (20%), blood (0%)  EN (34%), blood (NA) | *KRAS*: c.35G>T, p.Gly12Val  *KRAS:* c.35G>A, p.Gly12Asp | Chang et al, 2021 |
| 36 | M | 7 months | PPK, **embryonal rhabdomyosarcoma** | EN (8%), rhabdomyosarcoma (30-50%) | *HRAS:* c.37G>C, p.Gly13Arg | Davies et al, 2022 |
| 37 | M | 4 yo | Schimmelpenning–Feuerstein–Mims syndrome, **embryonal rhabdomyosarcoma** | NS (39.9%), ERMS (73.03%), blood (14.16%), non-lesional skin (8.65%) | *HRAS:* c.38G>T (p.Gly13Val) | Luo et al, 2021 |
| 38 | M | 4 yo | PPK | EN (21%), NS (20%), hair follicle (0%), normal skin (0%), blood (0%) | *HRAS:*c.181 C>A (p.Gln61Lys) | Huang et al, 2022 |

EN: Epidermal Nevus; UCC: Urothelial-Cell Carcinoma; PPK: Phacomatosis pigmento-keratotica; KEN: Keratinocit Epidermal Nevus; SK: Seborrheic keratosis; NA: Not Available; pt: patient; SCP: Syringocystadenoma papilliferum; TAA: Tubular apocrine adenoma; SLN: speckled lentiginous nevus; SCC: squamous cell carcinomas
